# Quantitative Analysis of miRNA Expression in Seven Human Foetal and Adult Organs

**DOI:** 10.1371/journal.pone.0028730

**Published:** 2011-12-14

**Authors:** Yanping Tang, Dong Liu, Lijie Zhang, Sigurdur Ingvarsson, Huiping Chen

**Affiliations:** 1 Department of Medical Genetics, Tongji Medical College, Huazhong University of Science and Technology, Hubei, China; 2 Department of Blood Transfusion, Wuhan Blood Centre, Hubei, China; 3 Institute for Experimental Pathology, Faculty of Medicine, University of Iceland, Reykjavik, Iceland; University of Georgia, United States of America

## Abstract

miRNAs have been found to repress gene expression at posttranscriptional level in cells. Studies have shown that expression of miRNAs is tissue-specific and developmental-stage-specific. The mechanism behind this could be explained by miRNA pathways. In this study, totally 54 miRNAs were analysed in 7 matched human foetal and adult organs (brain, colon, heart, kidney, liver, lung and spleen) using real-time PCR. Quantitative analysis showed that a big proportion of the 54 miRNAs have higher general expression in the organs of the foetal period than the adult period, with the exception of the heart. The miRNA gene promoter methylation level in the adult stages was higher than in the foetal stages. Moreover, there is a high general expression level of several miRNAs in both stages of brain, kidney, liver, lung and spleen, but not seen in colon and heart. Our results indicate that the miRNAs may play a bigger role in the foetal stage than the adult stage of brain, colon, kidney, liver, lung and spleen. The majority of the miRNAs analysed may play an important role in the growth and development of brain, kidney, liver, lung and spleen. However, a minority of the miRNAs may be functional in colon and heart.

## Introduction

The expression inhibition of genes can be generated by endogenous microRNAs (miRNAs). The miRNAs are non-coding RNAs that inhibit translations of target mRNAs or cleave the target mRNAs. The primary miRNAs (pri-miRNAs) are transcripts of the miRNA genes in the genome. The pri-miRNAs are turned into approximate 70 nucleotides of hairpin structures, called precursor miRNAs (pre-miRNAs), by Drosha, in the nucleus. The pre-miRNAs are then transported to the cytoplasm by Exportin-5 and are cleaved to about 22 nucleotides of mature miRNAs by Dicer enzymes [1].

Previous studies show that miRNAs are involved in some biological processes. Let-7 and lin-4 regulate the timing of early and late larval developmental transition in *Caenorhabditis elegans* [2], [3]. Some miRNAs play a role in flowering, leaf development and embryonic patterning in plants [4]–[6]. Moreover, in *Drosophila*, miR-14 and bantam are found to be a key regulator in cell apoptosis and growth and fat metabolism [7], [8]. It has been shown that the miRNAs are involved in development and differentiation of human cells [8]–[12]. Furthermore, miRNAs exhibit tissue-specific and developmental-stage-specific expression [13], [14]. In this study seven organs (brain, colon, heart, kidney, liver, lung and spleen) at foetal and adult stage were studied for miRNA expression. The 7 organs are crucial for the human body and have multiple functions. It can be speculated that a special group of miRNAs may be involved in regulation of function and dysfunction, differentiation, growth and development of these organs.

To date, a few articles have reported miRNA identification in human foetal and adult organs [15]–[18]. More work is necessary to gain an overview of expression of the miRNAs during the process of organ growth and development. Here we chose 54 miRNAs for quantitative analysis, of which the 31 were identified from the foetal livers in our previous studies [17], [18]. The rest were chosen from the miRNA database. Expression of the 54 miRNAs were tissue specific and involved in the growth and development of cells and tumorigenesis according to the miRNA database and literatures. We then quantified those miRNAs in the 7 matched human foetal and adult organ tissues using real-time PCR.

## Results

### The miRNA expression in the 7 matched human organs

**Figure 1:**
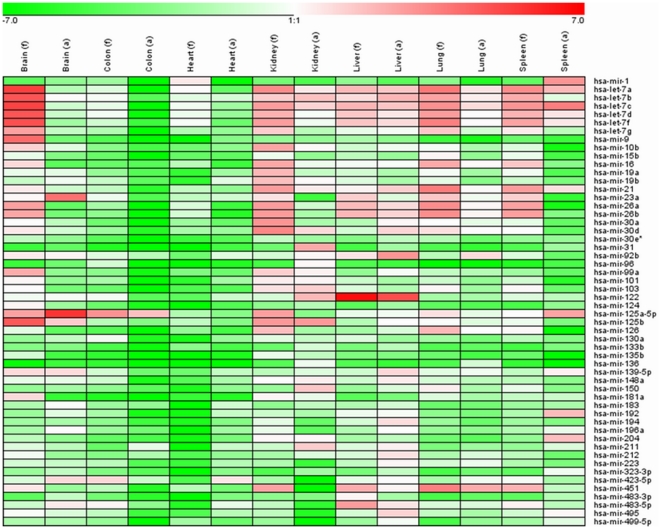
Expression profiles of 54 mature miRNAs in the 7 matched organ samples. Samples and miRNAs are displayed in rows and columns, respectively. The relative expression values ranged from +7 log_2_ to −7 log_2_, which are exhibited with different colors. The bright red colors represent the highest expression levels, the light green colors represent the lowest expression levels. f, foetal; a, adult.

In order to understand whether the expression level of the miRNAs differs in different stages and different organs, a quantitative PCR was performed. The relative expression level was calculated and compared (Fig. 1 and Table 1). A high level of miR-1 was identified in the adult spleen, and however a moderate level of miR-1 was seen in the foetal heart. The let-7 family (7a, 7b, 7c, 7d, 7f and 7g) showed a high level expression in the foetal brain. The high level of miR-9 and miR-125b was also detected in the foetal brain. In contrast, the miR-23a and miR-125a-5p exhibited the high level in the adult brain. The miR-21 was expressed highly in the foetal lung, spleen and kidney. A high level of miR-26a and miR-26b was identified in the foetal lung, kidney and spleen. The miR-122 exhibited the highest level in both foetal and adult liver. Also the highest level of miR-125a-5p was identified in both foetal and adult colon. The miR-451 was highly expressed in the foetal and adult lung, foetal kidney, liver and spleen.

**Table 1:** The top 10 expression level of miRNAs in each organ.

Fetal Brain	Fetal Colon	Fetal Heart	Fetal kindey	Fetal Liver	Fetal Lung	Fetal Spleen
hsa-let-7a	hsa-mir-125a-5p	hsa-mir-1	hsa-mir-30d	hsa-mir-122	hsa-let-7a	hsa-let-7a
hsa-let-7c	hsa-mir-423-5p	hsa-let-7a	hsa-let-7a	hsa-mir-483-5p	hsa-mir-21	hsa-let-7c
hsa-let-7f	hsa-mir-483-5p	hsa-let-7f	hsa-mir-30a	hsa-mir-451	hsa-let-7f	hsa-mir-21
hsa-let-7b	hsa-let-7b	hsa-let-7c	hsa-mir-125b	hsa-let-7c	hsa-mir-451	hsa-mir-26b
hsa-let-7d	hsa-let-7d	hsa-let-7d	hsa-mir-26b	hsa-mir-26b	hsa-mir-26a	hsa-mir-26a
hsa-mir-125b	hsa-mir-122	hsa-let-7b	hsa-mir-21	hsa-let-7a	hsa-mir-26b	hsa-let-7f
hsa-mir-9	hsa-mir-130a	hsa-mir-26a	hsa-mir-26a	hsa-mir-26a	hsa-let-7c	hsa-let-7b
hsa-mir-26a	hsa-mir-23a	hsa-mir-26b	hsa-let-7f	hsa-let-7f	hsa-let-7d	hsa-let-7d
hsa-let-7g	hsa-let-7c	hsa-mir-21	hsa-let-7c	hsa-let-7d	hsa-let-7b	hsa-mir-451
hsa-mir-125a-5p	hsa-mir-92b	hsa-mir-23a	hsa-mir-125a-5p	hsa-mir-23a	hsa-mir-16	hsa-mir-16

Adult Brain	Adult Colon	Adult Heart	Adult Kidney	Adult Liver	Adult Lung	Adult Spleen
hsa-mir-125a-5p	hsa-mir-125a-5p	hsa-mir-423-5p	hsa-mir-125b	hsa-mir-122	hsa-mir-451	hsa-let-7c
hsa-mir-23a	hsa-mir-211	hsa-mir-483-5p	hsa-mir-31	hsa-mir-92b	hsa-mir-92b	hsa-mir-1
hsa-mir-125b	hsa-mir-423-5p	hsa-mir-19b	hsa-mir-122	hsa-let-7b	hsa-let-7c	hsa-mir-125a-5p
hsa-mir-423-5p	hsa-mir-92b	hsa-mir-92b	hsa-let-7b	hsa-let-7c	hsa-let-7a	hsa-let-7a
hsa-mir-139-5p	hsa-mir-212	hsa-let-7b	hsa-mir-150	hsa-let-7a	hsa-let-7b	hsa-mir-192
hsa-mir-483-5p	hsa-mir-183	hsa-mir-23a	hsa-mir-211	hsa-mir-21	hsa-mir-26b	hsa-mir-204
hsa-mir-92b	hsa-mir-483-5p	hsa-mir-183	hsa-mir-103	hsa-mir-495	hsa-mir-26a	hsa-mir-21
hsa-let-7b	hsa-mir-125b	hsa-mir-122	hsa-let-7c	hsa-mir-26b	hsa-mir-21	hsa-let-7d
hsa-mir-196a	hsa-mir-196a	hsa-mir-211	hsa-mir-30d	hsa-mir-30d	hsa-mir-16	hsa-let-7f
hsa-mir-183	hsa-mir-139-5p	hsa-mir-192	hsa-let-7a	hsa-mir-139-5p	hsa-let-7f	hsa-mir-139-5p

### Comparison of the total miRNA expression in the 2 stages

**Figure 2:**
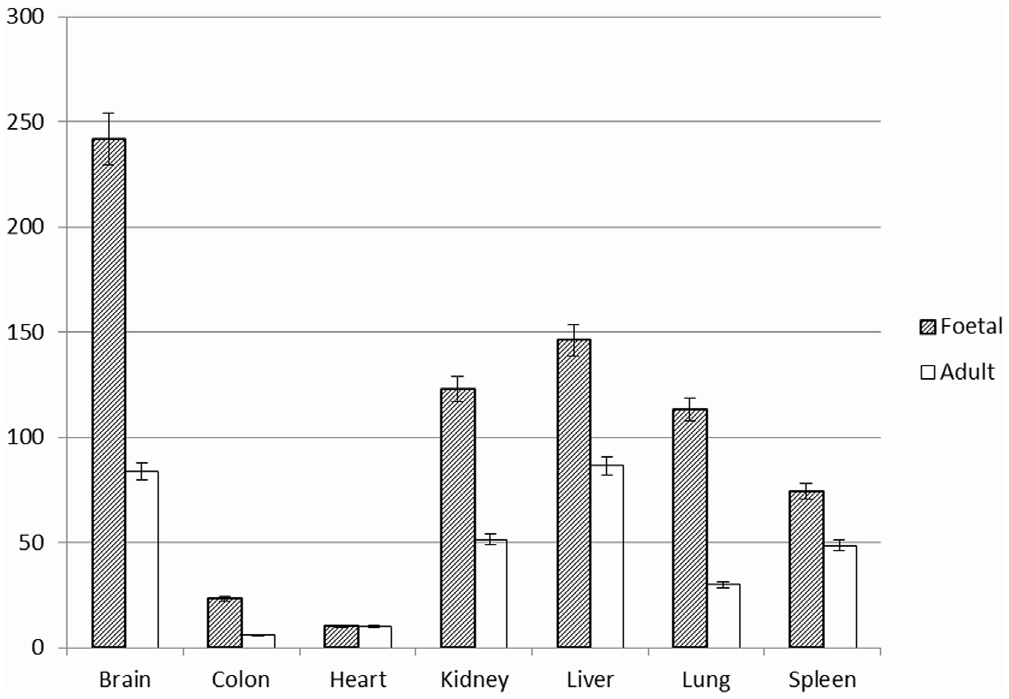
Comparison of the total expression levels of all 54 miRNAs in the seven matched organ tissues (addition of the relative expression level for each miRNA). The levels at the foetal stage are significantly higher than at the adult stages except the heart organs.

The total expression level of the miRNAs at each stage was calculated and compared (Fig. 2). The total level for 6 organs (brain, colon, kidney, liver, lung and spleen) at the foetal stage was significantly higher than at the adult stage (all p<0.001). No difference was observed between the foetal and adult stages for the heart organ (p = 0.78).

### Comparison of the total miRNA expression in the 7 organs

For the 7 foetal organs, the highest total miRNA expression level was seen in brain. The moderate total expression level was identified in the organs of kidney, liver, lung and spleen. The colon and heart organs exhibited lower total expression level (Fig. 2). For the 7 adult organs, higher total expression level was seen in the brain and liver organs. The moderate total expression level was detected in the kidney, lung and spleen organs. The colon and heart organs also showed lower total expression level compared to the foetal stage (Fig. 2).

### Comparison of miRNA gene methylation level in the 2 stages

**Figure 3:**
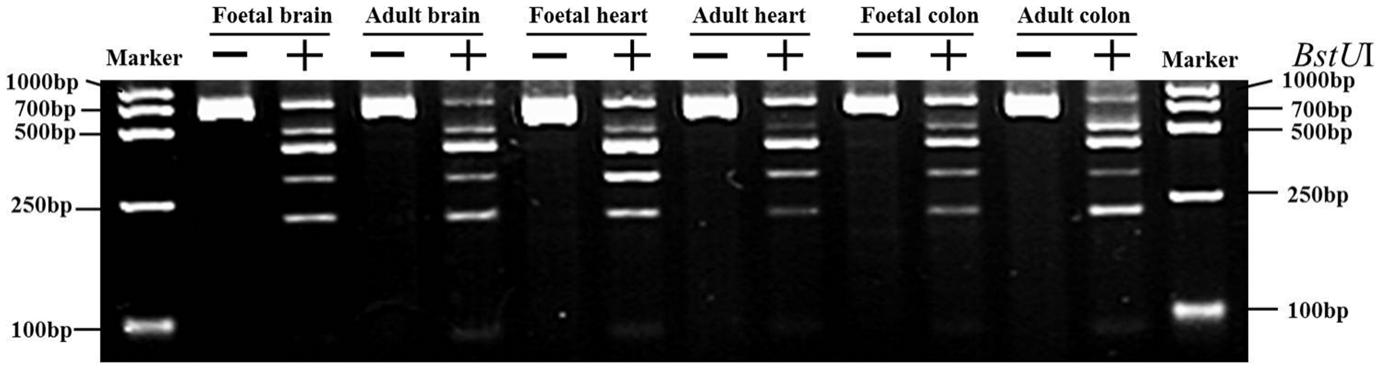
Promoter methylation assessment of let-7a gene by COBRA with *BstU*I. The foetal and adult brains, hearts and colons were analysed. Symbol “+” means that PCR products (723 bp) were digested with *BstUI*, and “−” means digestion without *BstUI*.

The gene promoter methylation of let-7a was examined in the foetal and adult brain, heart and colon organs (Fig. 3). The methylation level was calculated. For foetal and adult brains, the scores were 66% and 85% respectlvely. The foetal and adult colons exhibited 70% and 86% respectively. Also, 73% and 78% were for foetal and adult hearts respectively. In concordance, the expression level for the let-7a in the foetal brain was 144 fold that in the adult brain. For the colon organs, the ratio between foetal and adult stages was 119, and in the hearts, it was 20.

## Discussion

Gene expression exhibits tissue and developmental-stage specificity. The mechanism behind this could be that epigenetic events, including methylation modification and miRNAs actions, regulate transcription and translation of genes at certain tissues and developmental stages. In this study we analysed expression level of 54 miRNAs in 7 matched human organ tissues. These tissues were from 2 different stages of development: one was 30 weeks of foetal stage and another was 18 years of adult stage.

In general the total expression level of the 54 miRNAs was higher at the foetal stage than at the adult stage, except for the heart (Fig. 2). This is in line with our previous findings in the liver [18]. It indicates that the target gene functions are repressed at the foetal stage. At this stage, the embryo cells are in the process of differentiation. It is conceivable that the highly expressed miRNAs could be involved in embryo differentiation. The mechanism behind this difference in expression of miRNA between the foetal and adult stage is unknown, but one explanation could be higher gene methylation status in the adult [19]. We analysed and compared the gene promoter methylation level of let-7a, and found that the methylation level in the adult stages was higher than in the foetal stages (Fig. 3). In addition to transcriptional control and other mechanism of gene regulation, it can be inferred that at the foetal stage the inhibition of gene functions are mainly induced by the miRNAs, while in adults gene methylation plays a bigger role. No difference was seen for the total expression level between the foetal and adult stage of heart, and hearts at both stage showed very low level of miRNA expression compared to other organs (Fig. 2). This suggests that few miRNAs are implicated in the growth and development of the foetal heart at the stage of 30 weeks. It has been demonstrated that human cardiac development spans 6 weeks to 9 weeks of the foetal stage [20]. Thus, at the stage of 30 weeks, a low level of the general miRNA expression was seen, probably owing to the completed cardiac development. Surprisingly, the total expression level in the foetal brain was much higher than in the other foetal organs (Fig. 2), suggesting that those miRNAs play a big role in the growth and development of the foetal brain.

The miRNAs whose expression level ranks top 10 in each organ were analysed (Table 1). Involvement of different miRNAs can be seen in the foetal and adult organs. It indicates that different miRNAs play a role in the foetal and adult organs respectively. However, some miRNAs were involved in both foetal and adult stages, suggesting that they are conservative during the cell growth and development. For the brain, the let-7 family seems to play a bigger role at the foetal stage, however the miR-125a-5p and miR-23a do at the adult stage. In the foetal heart, miR-1 showed a highest level, however, the highest level is for miR-423-5p in the adult heart. The miR-30d and miR-125b display the highest level at the foetal and adult stages of kidneys, respectively. In contrast, the miR-122 was expressed highly in both foetal and adult liver. Also, the miR-451 displayed high expression level in both foetal and adult lungs.

Interestingly, the let-7 family was identified at a moderate to high level in the all foetal organs (Fig. 1 and Table 1). Members of the let-7 family can act as tumour suppressors and play a critical role in cell-cycle control with respect to differentiation and tumourigenesis [21]. The moderate to high expression of the let-7 family detected in the foetal organs suggests a certain level of negative regulation of cell growth. It has been reported that miR-125a-5p inhibits the proliferation of human gastric cancer cells in combination with trastuzumab [22]. The miR-23a promotes the growth of gastric adenocarcinoma cells [23]. Thus both miR-125a-5p and miR-23a can regulate cell growth.

The relatively high expression of miR-1 and mir-423-5p that we detected in foetal and adult hearth, respectively, is of interest. A previous study shows that miR-1 regulates differentiation and proliferation in human-derived cardiomyocyte progenitor cells [24]. Also, it seems that the miR-423-5p can act as a circulating biomarker for heart failure [25]. Hence both miR-1 and miR-423-5p are linked to establishment of structure and function of heart.

The miR-30d was found to promote tumor invasion and metastasis in hepatocellular carcinoma [26]. In contrast, the miR-125b functions as a tumor suppressor in invasive breast cancer [27]. Therefore both miRNAs are involved in the tumour invasiveness. The liver-specific miR-122 displayed moderate to high expression levels in the foetal and adult brain, foetal colon, adult kidney, and foetal and adult liver (Fig. 1). The target genes of miR-122 include PINX1_HUMAN. This gene may inhibit cell proliferation and act as a tumour suppressor. It has been suggested that inhibition of this gene may promote cell growth [28]. Studies uncovered that the miR-451 functions as a tumor suppressor in human non-small cell lung cancer and glioma cells [29], [30]. The high level of miR-451 detected in the foetal and adult lung, foetal kidney, liver and spleen, indicates that miR-451 plays a role as a negative regulator in the growth and development of those organs.

In conclusion the expression pattern of the analysed miRNAs is both organ and stage specific. The majority of the 54 miRNAs may play a bigger role in the foetal stage than the adult stage of brain, colon, kidney, liver, lung and spleen. These miRNAs may be involved in the growth and development of brain, kidney, liver, lung and spleen. However, a minority of them may regulate their target genes in colon and heart.

## Materials and Methods

### Ethics Statement

This research has been approved by the review board of Huazhong University of Science and Technology. We obtained tissue samples with written informed consent from the participants involved in the study. The ethics committee of Huazhong University of Science and Technology specifically approved the procedures.

### Samples

The 7 foetal organ tissues were obtained from a female foetus of 30 weeks, delivered due to an adenoma in the right lung, in Tongji hospital, Wuhan, Hubei, China. The fetus died shortly after it was delivered. The lung tissue obtained was from the left normal lung. The mother of the foetus was a healthy individual. The 7 adult organ tissues were obtained from a female aged 18 years, who had died due to severe injury, at the Department of Forensic Science, Tongji Medical College, Huazhong University of Science and Technology.

### Isolation of small RNA

Small RNAs (≤200 nt) were isolated from the matched 7 organ tissues using a mirVana™ miRNA isolation kit (Ambion, Austin, TX) following the manufacturer's instructions. About fifty milligrams of tissue were used and the small RNA was eluted in 100 µl RNAse-free water. The RNA concentration was tested by UV absorbance at 260 nm.

### Establishment of cDNA library

The small RNAs were polyadenylated at 37°C for 30 min in 50 µl reaction volume using ∼1 µg RNA and 5 U poly(A) polymerase (New England Biolabs). Then the poly(A)-tailed small RNA was purified through phenol/chloroform extraction and ethanol precipitation. Reverse transcription was conducted using the entire poly(A)-tailed RNA and 1 µg RT primer (5′-CGC TAC GTA ACG GCA TGA CAG TG(T)24-3′) with 200 U of SuperScript III reverse transcriptase (Invitrogen) according to the manufacturer's instructions.

### miRNA quantitative analysis using real-time PCR

Real-time PCR was carried out in a Stratagene MX-4000 system with the miRNA-specific forward primers (Table S1) and reverse primer (5′-CGC TAC GTA ACG GCA TGA CAG TG-3′) , and TransStart SYBR green qPCR supermix (TransGen, Biotech) following the manufacturer's instructions. The 20 µl reactions including 0.5 µl of RT products, 1× TransStart SYBR green qPCR supermix and 0.5 µM forward and reverse primers were incubated at 95°C for 5 min, followed by 40 cycles of 95°C for 10 sec, 55°C for 15 sec and 72°C for 20 sec. Melting curves for each PCR were carefully monitored to avoid non-specific amplification. The U6 small nuclear RNA was used as internal control. Each miRNA was analysed in duplicate. The relative level (RL) of each miRNA expression was calculated with 2^−ΔCt^ method, and the data were presented as log_2_ of RL of target miRNAs. Results were visualised with GENESIS (Alexander Sturn, Institute for Genomics and Bioinformatics, Graz University of Technology).

### DNA isolation and miRNA gene methylation assessment

For the foetal and adult brain, heart and colon tissues, DNA was extracted using DNA isolation kit (Sangon, Shanghai, China). The extracted DNA was converted using EZ DNA Methylation Kit (Zymo Research Corporation, Orange, CA, USA). The promoter methylation of the let-7a gene was assessed using combined bisulfite restriction analysis (COBRA) with BstUI [31]. The intensity of the bands detected by COBRA was examined by using QUANTITY ONE 4.62 (Bio-Rad). The methylation level was determined by calculating the intensity percentages of the 4 smaller digestion bands (Fig. 3).

### Statistical analysis of miRNA expression

A two-tailed *t*-test was used to assess the miRNA expression level difference at the two stages.

## Supporting Information

Table S1 **Fifty-four miRNAs specific primers for real-time PCR.**
(DOC)
