# Supplementary material for: Quantitative Analysis of miRNA Expression in Seven Human Foetal and Adult Organs
Source: PLoS One. 2011 Dec 14;6(12):e28730. doi: 10.1371/journal.pone.0028730 (PMC3237490; doi:10.1371/journal.pone.0028730)
Supplement: Table S1 — Fifty-four miRNAs specific primers for real-time PCR. (DOC) [file pone.0028730.s001.doc]

**Table S1. Fifty-four miRNAs specific primers for real-time PCR**

| miRNA | Primer（5’-3’） |
| --- | --- |
| hsa-mir-1 | TGGAATGTAAAGAAGTATGTAT |
| hsa-let-7a | TGAGGTAGTAGGTTGTATAGT |
| hsa-let-7b | TGAGGTAGTAGGTTGTGTGGTT |
| hsa-let-7c | TGAGGTAGTAGGTTGTATGGTT |
| hsa-let-7d | AGAGGTAGTAGGTTGCATAGTT |
| hsa-let-7f | TGAGGTAGTAGATTGTATAGTT |
| hsa-let-7g | TGAGGTAGTAGATTGTATAGTT |
| hsa-miR-9 | TCTTTGGTTATCTAGCTGTATGA |
| hsa-miR-10b | TACCCTGTAGAACCGAATTTGTG |
| has-miR-15b | TAGCAGCACATCATGGTTTAC |
| hsa-miR-16 | TAGCAGCACGTAAATATTGGCG |
| hsa-miR-19a | TGTGCAAATCTATGCAAAACTG |
| hsa-miR-19b | TGTGCAAATCCATGCAAAACTG |
| hsa-miR-21 | TAGCTTATCAGACTGATGTTG |
| hsa-miR-23a | ATCACATTGCCAGGGATTTCC |
| hsa-miR-26a | TTCAAGTAATCCAGGATAGG |
| hsa-miR-26b | TTCAAGTAATTCAGGATAG |
| hsa-miR-30a | TGTAAACATCCTCGACTGGAAG |
| hsa-miR-30d | TGTAAACATCCCCGACTGGAAG |
| hsa-miR-30e* | CTTTCAGTCGGATGTTTAC |
| hsa-miR-31 | AGGCAAGATGCTGGCATAGCT |
| hsa-miR-92b | TATTGCACTCGTCCCGGCCTCC |
| hsa-miR-96 | TTTGGCACTAGCACATTTTTGCT |
| hsa-miR-99a | AACCCGTAGATCCGATCTTGTG |
| hsa-miR-101 | TACAGTACTGTGATAACTGAA |
| hsa-miR-103 | AGCAGCATTGTACAGGGCTATG |
| hsa-miR-122 | TGGAGTGTGACAATGGTGTTTG |
| hsa-miR-124 | TAAGGCACGCGGTGAATGCC |
| hsa-miR-125a-5p | TCCCTGAGACCCTTTAACCTGT |
| hsa-miR-125b | TCCCTGAGACCCTAACTTGT |
| hsa-miR-126 | TCGTACCGTGAGTAATAATG |
| has-miR-130a | CAGTGCAATGTTAAAAGGGCAT |
| hsa-miR-133b | TTTGGTCCCCTTCAACCAGCTA |
| hsa-miR-135b | TATGGCTTTTCATTCCTATGTGA |
| hsa-miR-136 | ACTCCATTTGTTTTGATGATGGA |
| hsa-miR-139-5p | TCTACAGTGCACGTGTCTCCAG |
| hsa-miR-148a | TCAGTGCACTACAGAACTTTGT |
| hsa-miR-150 | TCTCCCAACCCTTGTACCAGTG |
| hsa-miR-181a | AACATTCAACGCTGTCGGTGAGT |
| hsa-miR-183 | TATGGCACTGGTAGAATTCACT |
| hsa-miR-192 | CTGACCTATGAATTGACAGC |
| hsa-miR-194 | TGTAACAGCAACTCCATGTGG |
| hsa-miR-196a | TAGGTAGTTTCATGTTGTTGGG |
| hsa-miR-204 | TTCCCTTTGTCATCCTATGCCT |
| hsa-miR-211 | TTCCCTTTGTCATCCTTCGCCT |
| hsa-miR-212 | TAACAGTCTCCAGTCACGGCC |
| hsa-miR-223 | TGTCAGTTTGTCAAATACCCCA |
| hsa-miR-323-3p | CACATTACACGGTCGACCTCT |
| hsa-miR-423-5p | TGAGGGGCAGAGAGCGAGACTTT |
| hsa-miR-451 | AAACCGTTACCATTACTGAGTT |
| hsa-miR-483-3p | TCACTCCTCTCCTCCCGTCTT |
| hsa-miR-483-5p | AAGACGGGAGGAAAGAAGGGAG |
| hsa-miR-495 | AAACAAACATGGTGCACTTCT |
| hsa-miR-499-5p | TTAAGACTTGCAGTGATGTTT |
